# Biocontrol of watermelon Fusarium wilt caused by *Fusarium oxysporum* f. sp. *niveum* with *Corallococcus* sp. EGB

**DOI:** 10.3389/fmicb.2026.1950692

**Published:** 2026-09-17

**Authors:** Xinjie Li, Mingze Gao, Zhitao Geng, Minxin Hou, Xiangmin Li, Zhoukun Li

**Affiliations:** 1 Key Laboratory of Agricultural Environmental Microbiology, Ministry of Agriculture and Rural Affairs, College of Life Sciences, Nanjing Agricultural University, Nanjing, China; 2 Nanjing Foreign Language School, Nanjing, China; 3 Nanjing Institute of Environmental Sciences, Ministry of Ecology and Environment, Nanjing, China

**Keywords:** biological control, *Corallococcus* sp. EGB, *Fusarium oxysporum* f. sp. *niveum*, root-zone bacterial community, watermelon Fusarium wilt

## Abstract

Watermelon Fusarium wilt is a severe soil-borne disease that threatens continuous watermelon production. Effective disease control depends on the differentiation of pathogen races, the accumulation of soil-borne inoculum, and disturbances in the soil bacterial community caused by continuous cropping. In this study, a pathogenic fungus was isolated from diseased watermelon plants in a continuously cropped field in Dongtai, Jiangsu Province, China. Morphological characterization, multigene phylogenetic analysis, and pathogenicity assays revealed that the pathogenic isolate belongs to *Fusarium oxysporum* f. sp. *niveum* (FON). The biocontrol efficacy of the predatory myxobacteria *Corallococcus* sp. EGB against FON was also evaluated under field conditions in Dongtai. Strain EGB maintained high target gene copy numbers in soil under consecutive cropping, which in turn reduced the FON target gene copy numbers and thereby lowered the incidence of fusarium wilt. Additionally, EGB application alters the composition of the root-zone soil bacterial community, shifts dominant taxa, and changes diversity patterns in the field experiments. These results indicate that EGB effectively suppresses watermelon Fusarium wilt caused by FON under field conditions, supporting the potential of EGB as a sustainable strategy for managing watermelon Fusarium wilt.

## 1 Introduction

Watermelon (*Citrullus lanatus*) is an economically significant crop grown worldwide, with China as the leading producer. According to the FAOSTAT database, global watermelon production reaches approximately 105.0 million tons in 2024. Of this total, China produces about 63.51 million tons, representing nearly 60.5% of the global yield (Food and Agriculture Organization of the United Nations, 2026). Dongtai, located in the Yangtze River Delta of Jiangsu Province, is an important watermelon-producing region in China. The favorable coastal climate, suitable soil conditions, and well-developed protected cultivation system jointly contributed to the establishment of an integrated watermelon industry covering seedling production, cultivation, grading, and marketing. Watermelon produced in Dongtai is recognized as a Geographical Indications Agro-product. However, the expansion of protected cultivation and prolonged continuous cropping have intensified continuous cropping obstacles. These challenges include outbreaks of soil-borne diseases, disruption of the soil microbial balance, poor plant growth, and declining yields (Zhang et al., 2015; Tong et al., 2025). Among these constraints, Fusarium wilt of watermelon, caused by *Fusarium oxysporum* f. sp. *niveum* (FON), is one of the most destructive soil-borne diseases affecting watermelon production (Zhang et al., 2015; Rahman et al., 2021). Infected plants usually show symptoms such as wilting, root browning, and vascular discoloration. In severe cases, the entire plant may die, leading to substantial losses in both yield and fruit quality (Zhang et al., 2015; Tong et al., 2025). In severely affected fields, Fusarium wilt causes total crop failure. This disease poses a significant barrier to continuous watermelon cultivation and threatens the sustainable development of the watermelon industry.

Disease control in agro-ecosystems relies on various management strategies (Zwetsloot et al., 2020). Watermelon Fusarium wilt is mainly managed through chemical control, breeding of disease-resistant varieties, agricultural management and soil biological regulation (Roberts et al., 2023; Tong et al., 2025). Although chemical treatments can temporarily reduce pathogen populations in soil, their effectiveness is limited. *Fusarium oxysporum* f. sp. *niveum* produces chlamydospores that persist for long periods and gradually accumulate under continuous cropping systems (Rahman et al., 2021). Repeated chemical applications can further disrupt the soil microbial balance, promote the emergence of fungicide-resistant pathogen populations, and increase environmental risks (Roberts et al., 2023; Nguyen et al., 2025). Breeding disease-resistant cultivars remains an important component of disease management. However, its effectiveness is challenged by the physiological race differentiation of FON. Race 3 is highly virulent and can infect several cultivars that are resistant to race 0, race 1, or race 2, significantly limiting the practical use of existing resistance resources (Wechter et al., 2012; Rahman et al., 2021; Roberts et al., 2023). Therefore, developing effective disease management strategies for watermelon Fusarium wilt caused by FON race 3 is urgently needed in watermelon cropping systems. Biological control has become a promising and sustainable method for managing soil-borne diseases. It offers clear advantages over chemical control, particularly regarding environmental compatibility and ecological safety (Bakker et al., 2013). Unlike conventional chemical treatments, microbial biocontrol agents can suppress pathogens through several complementary mechanisms, including competition for niches and nutrients, production of antimicrobial metabolites and hydrolytic enzymes, induction of host systemic resistance, and modulation of the rhizosphere microbiome (Muñoz-Dorado et al., 2016; Thiery and Kaimer, 2020). Several microbial groups, including *Bacillus*, *Pseudomonas*, and *Trichoderma*, have been extensively studied for controlling soil-borne pathogens. Their ability to suppress disease is often linked to the production of lipopeptide antibiotics, siderophores, and enzymes that degrade cell walls (Boro et al., 2022; Woo et al., 2023). Therefore, introducing and establishing functional microorganisms offers a promising strategy to reduce pathogen abundance and restore microbial balance in soils under continuous cropping systems.

Myxobacteria can prey on plant pathogens and regulate the rhizosphere microbiome (Ye et al., 2020; Zhang et al., 2026), which are continued to regarded as promising biocontrol agents (Xia et al., 2023; Zhang et al., 2023; Wang et al., 2025). The predatory myxobacterium *Corallococcus* sp. EGB has proven to be an effective biocontrol agent against soil-borne Fusarium wilt. In cucumber, solid- fermented EGB significantly reduced Fusarium wilt through predation-mediated regulation of the soil microbial community. This approach achieved a biocontrol efficacy of 79.6% in the field (Ye et al., 2020). These findings indicate that EGB could suppress Fusarium wilt through direct predation and microbiome-mediated disease suppression. However, it is still unclear whether EGB could effectively control Fusarium wilt in continuously cropped watermelon fields. The response of soil microorganisms in consecutive cropping systems to EGB predation also remains unexplored. In this study, we isolated a pathogenic Fusarium strain from diseased watermelon plants in a continuously cropped field in Dongtai. We characterized its taxonomic identity using differential-host and molecular marker assays. Next, we assessed the antagonistic activity and field biocontrol efficacy of EGB against this isolate. We also examined the effects of EGB on the soil bacterial community using high-throughput sequencing. This study aimed to provide a basis for developing a myxobacterium-based strategy for the biological control of watermelon Fusarium wilt and improving soil bacterial community management under continuous cropping conditions.

## 2 Materials and methods

### 2.1 Materials

Diseased watermelon samples were collected from symptomatic plants in watermelon fields in Wulie Town, Dongtai (32.8526°N, 120.2495°E‌). The watermelon cultivars sampled were “Shuangtian No. 6” and “Zaojia 8424”. The watermelon differential hosts, including “Crimson Sweet”, “Sugar Baby”, and “Calhoun Gray”, were kindly provided by the National Mid-term Genebank for Watermelon Germplasm Resources. *Corallococcus* sp. strain EGB (CCTCC M2012528), previously isolated from saline soil, was maintained in our laboratory at −80 °C in 15% (v/v) glycerol as a cryoprotectant. Before use, strain EGB was cultured on VY/4 agar medium (0.25% yeast cells, 0.1% CaCl₂, and 1.5% agar, pH 7.2, w/v) or in LBS liquid medium (0.5% yeast extract, 0.1% tryptone, and 0.7% soluble starch, pH 7.2, w/v). *Fusarium oxysporum* f. sp. *cucumerinum* (FOC) (ACCC 30220) was cultured on potato dextrose agar (PDA) plates.

### 2.2 Isolation and identification of the pathogen

Tissue samples were collected from the margins of lesions on symptomatic watermelon plants. The pathogen was isolated and purified as previously described, with minor modifications (Zhou et al., 2010). Small sections from the diseased–healthy vascular junction were surface-sterilized using 75% ethanol and sodium hypochlorite, then rinsed with sterile water and dried. These samples were incubated on potato dextrose agar at 25–28 °C. Fusarium-like colonies that emerged were transferred to fresh potato dextrose agar plates and purified through single-spore isolation. The resulting pure cultures were preserved for later morphological and molecular characterization, as well as pathogenicity assays. The fungal isolate was grown on PDA to evaluate colony morphology, pigmentation, mycelial growth, and other phenotypic traits.

Spores were collected by rinsing the mycelia with sterile water. Hyphae and spores were stained with lactophenol cotton blue (LPCB) and observed under a microscope. Genomic DNA from the pathogen was extracted using the CTAB method (Möller et al., 1992). The ITS, TEF-1α, and β-tubulin gene regions were amplified to identify the pathogen at the molecular level (Supplementary Table S1). PCR amplification followed the instructions provided by the DNA polymerase manufacturer. The PCR products were purified by electrophoresis on a 1.5% agarose gel and then sequenced. The resulting sequences were aligned with reference sequences from the NCBI database. Species-level identification was performed using these alignments along with phylogenetic analysis. The β-tubulin and TEF-1α gene sequences of FON DT0916 and the reference strains were aligned using ClustalW implemented in MEGA 11. Phylogenetic trees were constructed using the maximum-likelihood method with the Tamura–Nei substitution model. Evolutionary rates among sites were modeled using a gamma distribution with invariant sites (G + I) and five discrete gamma categories. Positions containing gaps or missing data were treated using partial deletion with a site coverage cutoff of 95%. Tree topology was optimized using the nearest-neighbor-interchange method, and branch support was evaluated using 1,000 bootstrap replicates.

The watermelon differential hosts “Crimson Sweet”, “Sugar Baby”, and “Calhoun Gray” were used to identify the physiological race of the pathogen, along with molecular marker assays. Seeds were treated according to the method described in Section 2.2. The pathogen was inoculated using the root-dip method with a conidial suspension at a concentration of 5 × 10^6^ conidia mL^−1^. For each differential host, three seedlings were planted in each pot, with four pots assigned to each treatment. Seedlings displaying uniform growth were selected. Their roots were immersed in the conidial suspension for 15 min. During inoculation, the suspension was gently shaken 4–5 times to prevent conidial sedimentation. After inoculation, the seedlings were transplanted into pots and maintained under the specified greenhouse conditions. Disease incidence was assessed after 15 days post-inoculation and calculated for each pot as follows: Disease incidence (%) = (number of symptomatic plants/total number of plants) × 100. The physiological race of FON DT0916 was identified based on the disease responses of the differential hosts and the amplification results of the W106, FOW, SIX6, and FNR3 molecular markers (Cao, 2016).

### 2.3 Pathogenicity test

The pathogenicity of the fungal isolate was tested on healthy, potted watermelon plants. Watermelon seeds were planted in potting substrate and kept in a greenhouse at 25–30 °C with a 16-h light and 8-h dark cycle. FON DT0916 was grown in potato dextrose broth to produce a conidial suspension. The conidial suspension was thoroughly mixed into the soil to achieve a final concentration of 5 × 10^6^ conidia per gram of soil. Uniform watermelon seedlings were then transplanted into the inoculated soil. Each treatment consisted of seven pots, with three watermelon plants per pot. For the uninoculated control, an equal volume of sterile water was added to the soil using the same procedure before transplanting the seedlings. Disease severity was assessed at 15 days post-inoculation (dpi) using a 0–4 scale: 0, no symptoms; 1, wilting of leaves or stems affecting 0–1/4 of the whole plant; 2, wilting affecting 1/4–1/2 of the whole plant; 3, wilting affecting 1/2–3/4 of the whole plant; and 4, the entire plant wilted or dead (Xu et al., 2004). The disease index (DI) was calculated separately for each pot according to the following equation:


$$\mathrm{DI}\;(\%)=[\begin{array}{l} \Sigma (\begin{array}{l} \text{disease rating}\times \text{number of plants}\;\\ \mathrm{at}\;\text{that rating} \end{array})\\ /(\begin{array}{l} \text{maximum disease rating}\times \text{total number}\\ \;\text{of plants} \end{array}) \end{array}]\times 100$$

Seeds of cucumber cultivar “Jinyan No. 4” were treated as previously described in Section 2.2. Once the cotyledons had fully expanded, uniformly growing seedlings were selected and transplanted into pots filled with pathogen-infested soil. Each treatment consisted of five pots with three plants per pot. Soil inoculation was carried out using *Fusarium oxysporum* f. sp. *cucumerinum* (FOC) and the isolated pathogen at a concentration of 5 × 10^6^ spores per gram of soil. An equal volume of sterile water was used as the control. Disease incidence was assessed at 21 days post-inoculation and calculated as follows: Disease incidence (%) = (number of symptomatic plants/total number of plants assessed) × 100. Disease responses were classified using the 0–5 scale as described before (Fareed et al., 2017): 0, immune (0% disease incidence); 1, resistant (>0–10%); 2, moderately resistant (11–25%); 3, moderately susceptible (26–45%); 4, susceptible (46–70%); and 5, highly susceptible (71–100%) (Fareed et al., 2017).

### 2.4 *Corallococcus* sp. EGB – FON confrontation assays

The predatory activity of EGB against FON DT0916 was assessed as described before (Li et al., 2017). EGB was co-cultured with FON DT0916 on VY/4 plates at 25 °C for 5 days, after which the colony diameter of FON DT0916 was measured. FON DT0916 without EGB incubation was used as control. The inhibition rate of EGB against FON mycelial growth was calculated as follows: inhibition rate (%) = [(colony diameter of FON in the control group − colony diameter of FON in the treatment group)/colony diameter of FON in the control group] × 100.

### 2.5 Field tests of watermelon Fusarium wilt biocontrol with *Corallococcus* sp. EGB

EGB was cultured on TPM agar plates to induce development and sporulation, and the resulting spores were collected and lyophilized to produce the EGB powder. Seedling responses to EGB were evaluated using 40 EGB-treated trays and 14 control trays, each containing 70 seeds. Three trays were randomly selected from each treatment for analysis, and each tray was treated as an independent experimental unit. Before sowing, EGB was incorporated into the growth substrate at a rate equivalent to 5 × 10^8^ CFU seedling^−1^. Germination percentage was recorded 15 days after sowing, EGB was applied at 5 × 10^8^ CFU seedling^−1^ on days 17 and 34 after sowing. Seedling height was measured 34 days after sowing, immediately before transplantation.

The field trial comprised seven plots (T1–T7) in second- and third-consecutive-cropping fields. Treatments included low- and high-dose EGB powder, EGB soil pretreatment combined with low-dose EGB, and untreated controls. In this study, soil pretreatment refers to the pre-application of EGB to the field soil to improve its initial distribution. Specifically, in T3, 35 g of the EGB preparation was thoroughly premixed with 50 kg of soil collected from the same plot, evenly distributed over the plot, and incorporated into the upper 20 cm of soil by rotary tillage. No chemical disinfectants, fungicides, fertilizers, or other exogenous amendments were used during pretreatment. During vine elongation, EGB was subsequently applied by drip irrigation at target rates of 1 × 10^5^ or 1 × 10^6^ CFU g^−1^ soil. Plot characteristics and detailed application procedures are provided (Table 1; Supplementary Tables S2–S5).

**Table 1:** Field experiment design.

Plot	Treatment
T1	Third consecutive cropping plot, low-concentration EGB treatment
T2	Third consecutive cropping plot, control blank control
T3	Third consecutive cropping plot, soil pretreatment + low-concentration EGB treatment
T4	Third consecutive cropping plot, high-concentration EGB treatment
T5	Second consecutive cropping plot, high-concentration EGB treatment
T6	Second consecutive cropping plot, low-concentration EGB treatment
T7	Second consecutive cropping plot, control blank control

Disease incidence was recorded cumulatively using the total number of plants in each plot as the denominator, and plants previously classified as diseased remained counted as diseased in subsequent assessments. A plant was classified as diseased when it exhibited visible symptoms consistent with Fusarium wilt, including progressive leaf yellowing, wilting of leaves or stems, whole-plant collapse, or death (Seid et al., 2024). Fruit yield was recorded at harvest on May 12, 2025. Immediately after harvest, root-zone soil was collected from each plot for qPCR and bacterial community analyses. Copy numbers of EGB and FON target genes were quantified by qPCR using soil DNA extracted with the FastDNA^®^ SPIN Kit and purified with the DNA Clean-Up™ Kit. The reaction mixtures and thermal cycling conditions were provided in Supplementary Tables S6, S7.

### 2.6 High-throughput sequencing analysis of field soil bacterial community structure

Soil was collected from the watermelon root zone at a depth of 3.0–5.0 cm after removing the surface layer. For each group, nine subsamples of 0.5 g were taken and pooled in sets of three, resulting in three composite samples. Root zone soil samples from each treatment were then sent to Majorbio Bio-Pharm Technology Co., Ltd. for high-throughput sequencing and bioinformatic analysis. The bacterial 16S rRNA gene was amplified with barcode-tagged primers 515F and 907R. PCR products were confirmed by electrophoresis on a 2% agarose gel. After gel extraction, the products were purified and quantified using fluorometry. These purified products were then used to construct sequencing libraries. Paired-end sequencing was carried out on the Illumina NextSeq 2000 platform. Raw 16S rRNA gene sequences were processed using a 97% OTU-based workflow. As a supplementary comparison, the sequencing data were additionally processed using DADA2 to infer exact amplicon sequence variants (ASVs) without similarity-based OTU clustering. Taxonomic assignment of ASVs was performed against the SILVA 138.2 database. Alpha diversity, principal component analysis (PCA), and bacterial community composition at the phylum level were subsequently analyzed using the ASV dataset and compared with the corresponding results obtained from the 97% OTU-based analysis. Detailed bioinformatic procedures, software versions, parameters, and ecological classification criteria were provided in Supplementary Tables S8, S9. The raw high-throughput sequencing data generated in this study have been deposited in the NCBI Sequence Read Archive (SRA) under BioProject accession number PRJNA1513764.

### 2.7 Statistical analyses

Rarefaction curves and alpha-diversity indices were calculated using Mothur. Differences among treatments were evaluated using one-way analysis of variance (ANOVA), followed by Duncan’s multiple range test for *post hoc* comparisons. Data that did not meet the assumption of normality were analyzed using the Kruskal–Wallis test, followed by Dunn’s multiple-comparison test. Data are presented as the mean ± standard error of the mean (SEM). Statistical analyses were performed using SPSS. Differences were considered statistically significant at *p* < 0.05.

## 3 Results

### 3.1 Isolation and identification of fungal strain FON DT0916 from diseased watermelon stems

Through tissue culture and separation methods, we isolated a fungal strain FON DT0916 from the diseased watermelon stems. The morphological characteristics of FON DT0916 were then examined (Figures 1A–C). The isolate grew rapidly on PDA, forming circular colonies with abundant aerial mycelia and a loose, white, cotton-like texture. A pale pink to reddish area developed at the center of the colony, while the marginal mycelia extended outward in a radial pattern. Microscopic examination after LPCB staining revealed hyaline, distinctly branched, and septate hyphae. The conidia were mostly elliptical, ovoid, or short rod-shaped, with relatively blunt ends. These morphological features matched the typical characteristics of fungi belonging to member of *Fusarium* (Kleczewski and Egel, 2011; Petkar et al., 2019; Dutta et al., 2024).

**Figure 1:**
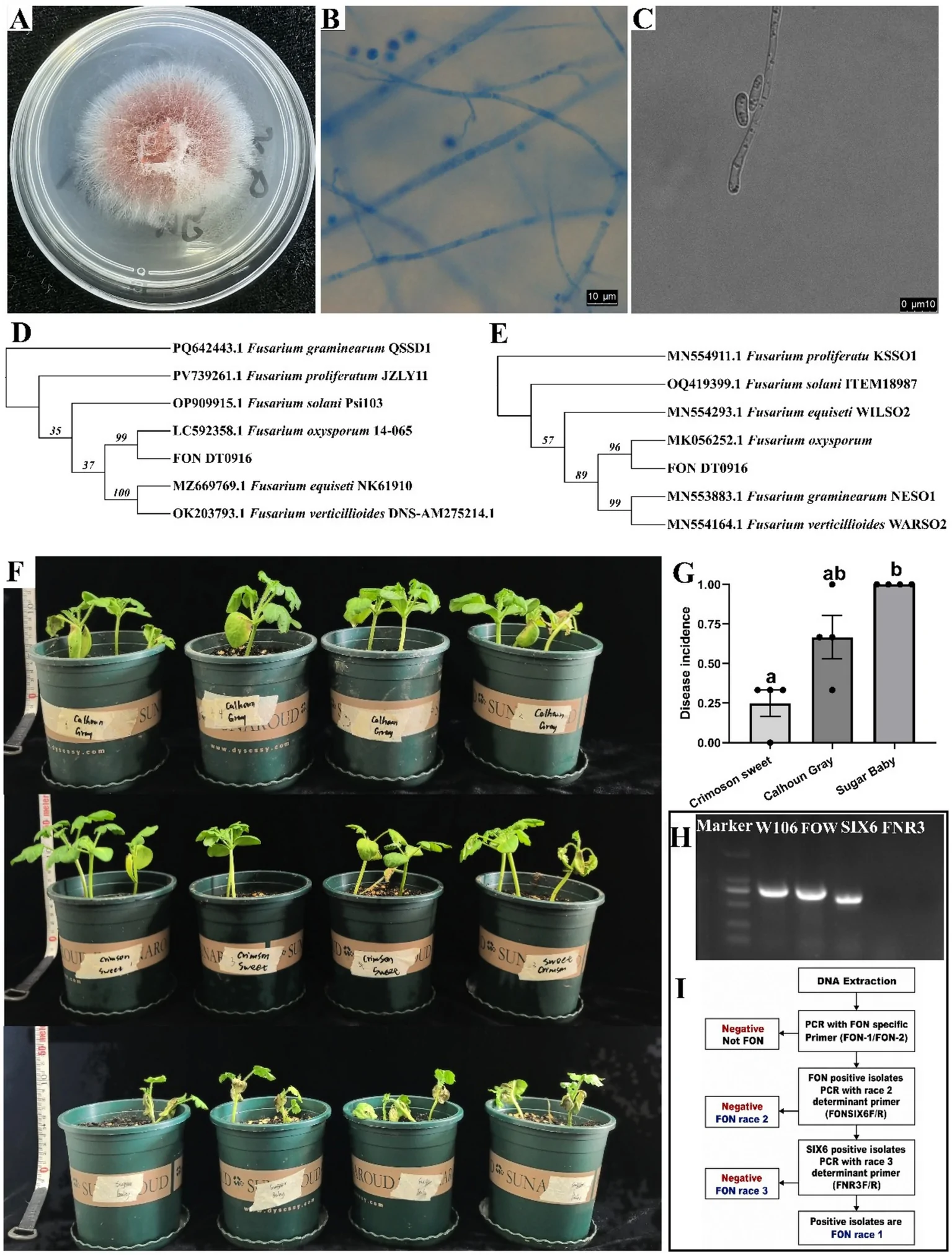
Morphological and molecular identification of isolated pathogenic fungi FON DT0916. **(A)** Colony morphology of FON DT0916. **(B)** Hyphal morphology after staining with lactophenol cotton blue. **(C)** Microscopic morphology of the conidia. **(D,E)** Maximum-likelihood phylogenetic trees inferred from β-tubulin **(D)** and TEF-1α **(E)** gene sequences. Sequences were aligned with ClustalW, and trees were constructed in MEGA 11 using the Tamura–Nei substitution model. Numbers at nodes represent bootstrap support values (%) calculated from 1,000 replicates. **(F)** Disease symptoms in the differential watermelon cultivars “Calhoun Gray”, “Crimson Sweet”, and “Sugar Baby” following inoculation with FON DT0916. **(G)** Disease incidence in the three cultivars following inoculation with FON DT0916. Bars represent the mean ± standard error of the mean (SEM), and dots represent individual replicate values. Different lowercase letters indicate significant differences among cultivars, as determined using the Kruskal–Wallis test followed by Dunn’s multiple-comparison test (*p* < 0.05). **(H)** PCR-based detection of the race-associated molecular markers W106, FOW, SIX6, and FNR3 in FON DT0916. **(I)** Flowchart illustrating the process for determining the physiological race of FON using molecular markers.

Phylogenetic trees were constructed using β-tubulin and TEF1-α sequences (Figures 1D,E). The TEF1-α (GenBank accession no. PZ871625) phylogeny placed FON DT0916 within the *Fusarium oxysporum* clade, supported by high bootstrap values. FON DT0916 was clearly separated from other Fusarium species, such as *F. solani, F. proliferatum, F. equiseti, F. verticillioides*, and *F. graminearum*. The β-tubulin (GenBank accession no. PZ871624) phylogenetic tree provided additional support for this classification. Combined with observations of colony morphology, microscopic features, and ITS sequence alignment (GenBank accession no. PZ841208), the isolate was identified as *Fusarium oxysporum*. The physiological race of FON DT0916 was determined through molecular marker analysis and inoculation assays on differential hosts. “Crimson Sweet” displayed relative resistance, with a disease incidence of 25.0%. In contrast, “Calhoun Gray” and “Sugar Baby” were susceptible, showing disease incidences of 66.7 and 100%, respectively (Figures 1F,G). PCR produced amplicons for W106, FOW, and SIX6, but not for FNR3. Considering the race-associated molecular marker results with the differential host reaction pattern, FON DT0916 was provisionally designated as a possible race 3 isolate of *Fusarium oxysporum* f. sp. *niveum* (Figures 1H,I).

### 3.2 Pathogenicity verification of strain FON DT0916

In the pathogenicity assay, at 15 dpi, plants inoculated with FON DT0916 developed typical Fusarium wilt symptoms (Figures 2A,B), and showed a mean disease index of 64.3 ± 9.6% (mean ± SEM, *n* = 7), whereas control plants remained asymptomatic (DI = 0) (Supplementary Table S10). FON DT0916 was re-isolated from symptomatic plants but was not recovered from the control group. Additionally, the ITS sequence of the re-isolated pathogen shared 100% sequence identity with the original FON DT0916 (Figure 2C). These results confirmed that the strain re-isolated from diseased watermelon plants is identical to FON DT0916, thereby fulfilling Koch’s postulates and validating the pathogenicity of FON DT0916.

**Figure 2:**
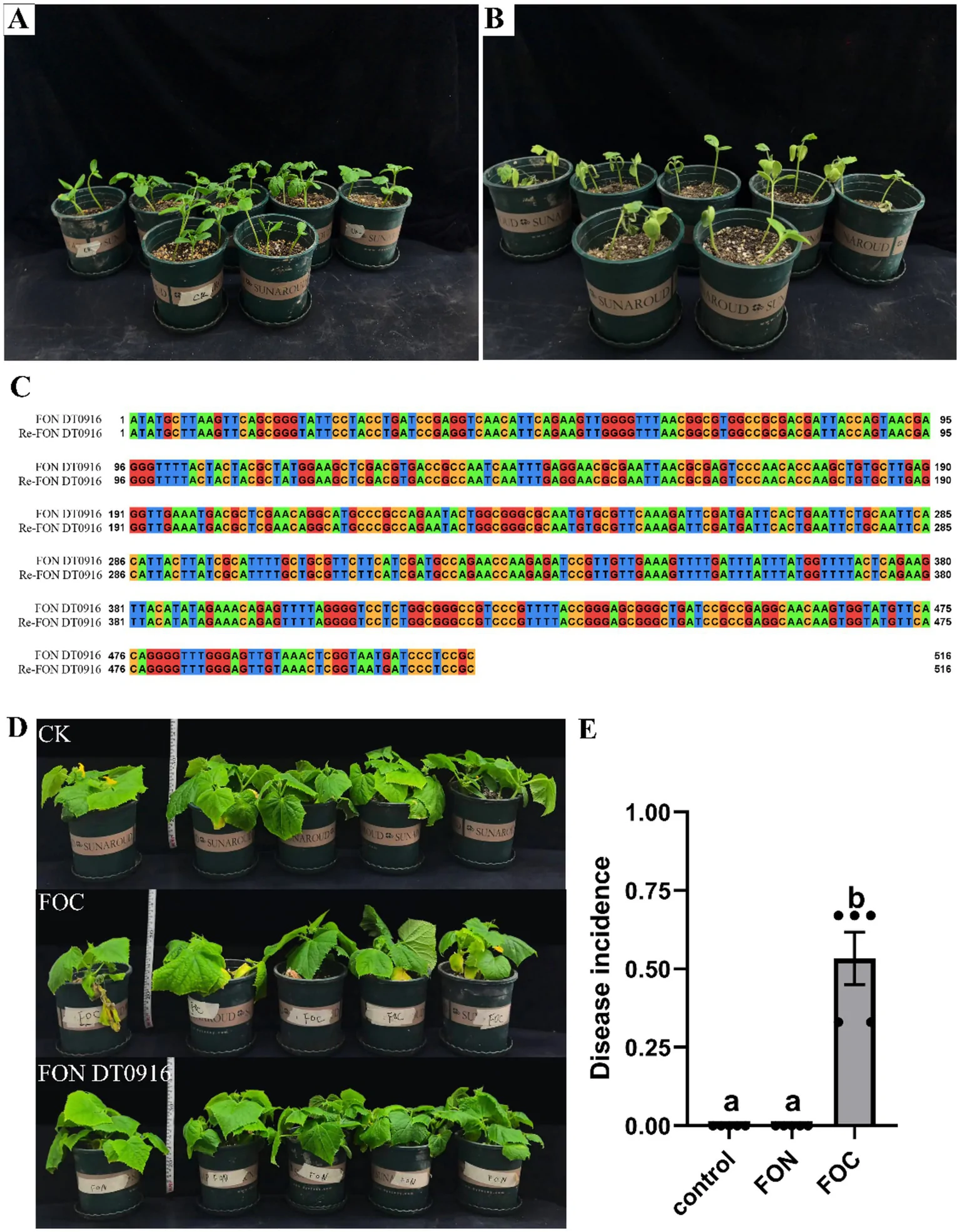
Pathogenicity assessment of FON DT0916. **(A)** Growth status of watermelon seedlings in the control group. **(B)** Disease symptoms of watermelon seedlings after inoculation with FON DT0916. **(C)** Pairwise alignment of ITS sequences between FON DT0916 and the re-isolated strain. **(D)** Growth and disease phenotypes of cucumber seedlings were evaluated under three treatments: control group, FOC-inoculated group, and FON DT0916-inoculated group. **(E)** Disease incidence of cucumber seedlings in the three treatment groups. Bars represent the mean ± SEM, and different lowercase letters indicate significant differences among treatments (*p* < 0.05).

We also evaluated the pathogenicity of FON DT0916 on cucumber, and *Fusarium oxysporum* f. sp. *cucumerinum* (FOC) responsible for cucumber Fusarium wilt was served as the positive control. After 21 days of inoculation, cucumber seedlings in the control group showed no signs of wilting or chlorosis. In contrast, plants inoculated with FOC exhibited wilting, leaf chlorosis, and inhibited growth, with a disease incidence of 53.4% (Figures 2D,E). Under this experimental conditions, FON DT0916 caused typical disease symptoms in watermelon but no symptoms in the tested cucumber cultivar.

### 3.3 *Corallococcus* sp. EGB inhibits growth of FON DT0916 by predation

The effect of EGB on the mycelial growth of FON DT0916 was assessed using a dual-culture assay. FON DT0916 showed rapid growth and significant radial mycelial expansion (Figure 3A). In contrast, the EGB-treated group displayed markedly restricted colony expansion, with a limited colony diameter (Figure 3B). The growth inhibition rate reached 62.77%. When EGB contacted with FON DT0916, the collapsed fungal colony was observed. The collapsed colony observed at the EGB–FON interaction zone suggests that EGB exerts a predatory effect on FON mycelium. This finding is consistent with our previous evidence that EGB uses β-1,6-glucanase to decompose fungal cell walls during predation (Li et al., 2019; Ye et al., 2020).

**Figure 3:**
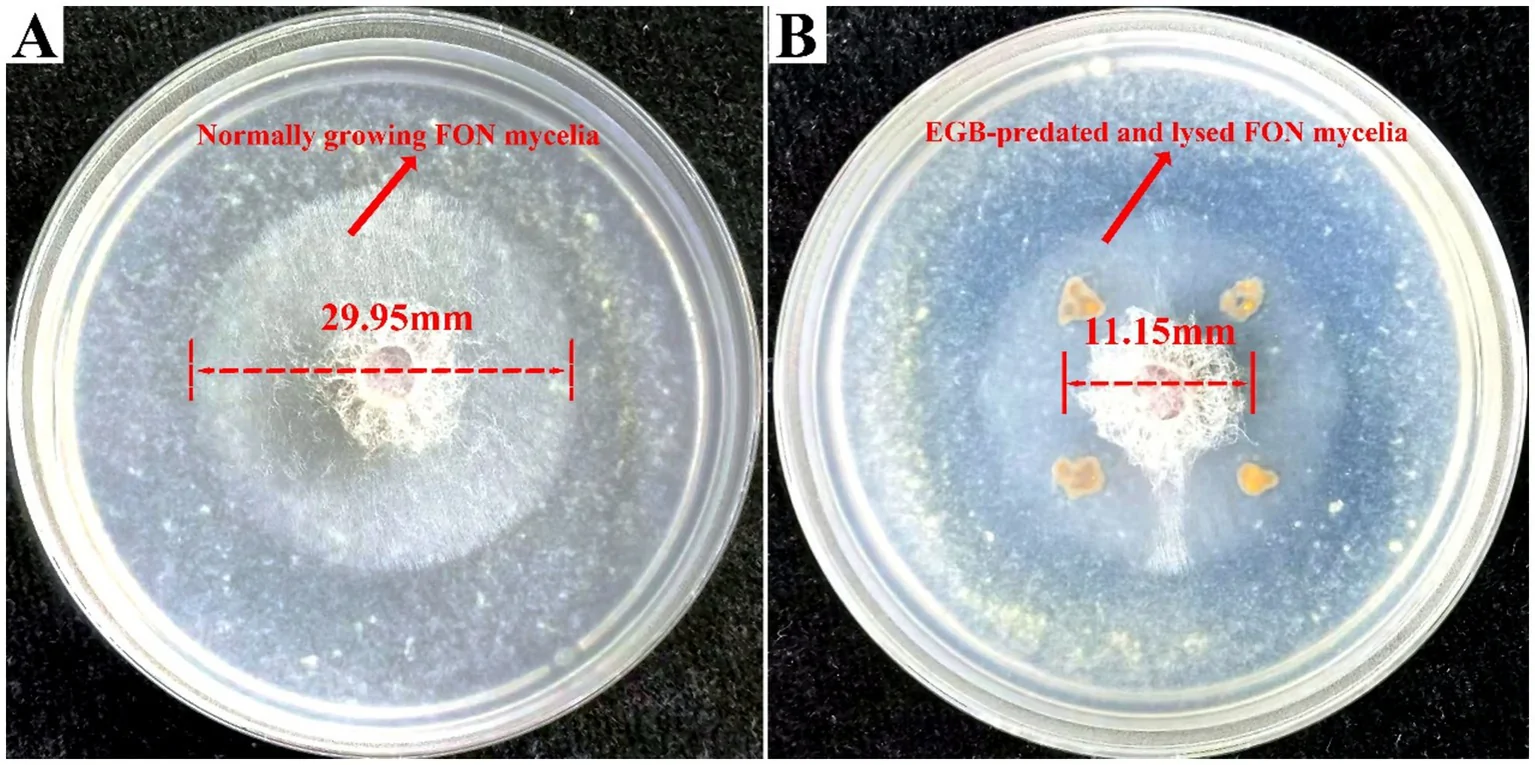
Growth inhibition of FON DT0916 by predatory EGB. **(A)** Pathogen control group without EGB inoculation. **(B)** Co-culture EGB with FON DT0916.

### 3.4 Field effects of EGB application on watermelon growth and fusarium wilt under continuous cropping condition

The effects of *Corallococcus* sp. EGB on watermelon growth and *Fusarium oxysporum* f. sp. *niveum* incidence were further evaluated under field conditions. Fifteen days after sowing, germination rates were significantly higher in the EGB treatment compared to the control (Figure 4A). At the time of transplanting, 34 days after sowing, EGB-treated seedlings were also taller than those in the control group (Figure 4B). Field observations indicated that plants in the EGB-treated plots showed better overall growth and greater canopy coverage than those in the control plots (Figures 4C,D). EGB treatment alleviated the inhibitory effects of continuous cropping on watermelon vine growth. In the third consecutive cropping control, main vine length was significantly reduced, whereas EGB-treated plots exhibited enhanced vine growth, with values comparable to those observed in the corresponding second consecutive cropping plots (Supplementary Figure S1). Disease incidence progressively increased in untreated control plots, whereas EGB-treated plots maintained substantially lower disease incidence throughout the investigation period (Figure 4E). Otherwise, EGB treatment enhanced yield even under continuous-cropping conditions (Figure 4F), indicating its potential to stabilize production.

**Figure 4:**
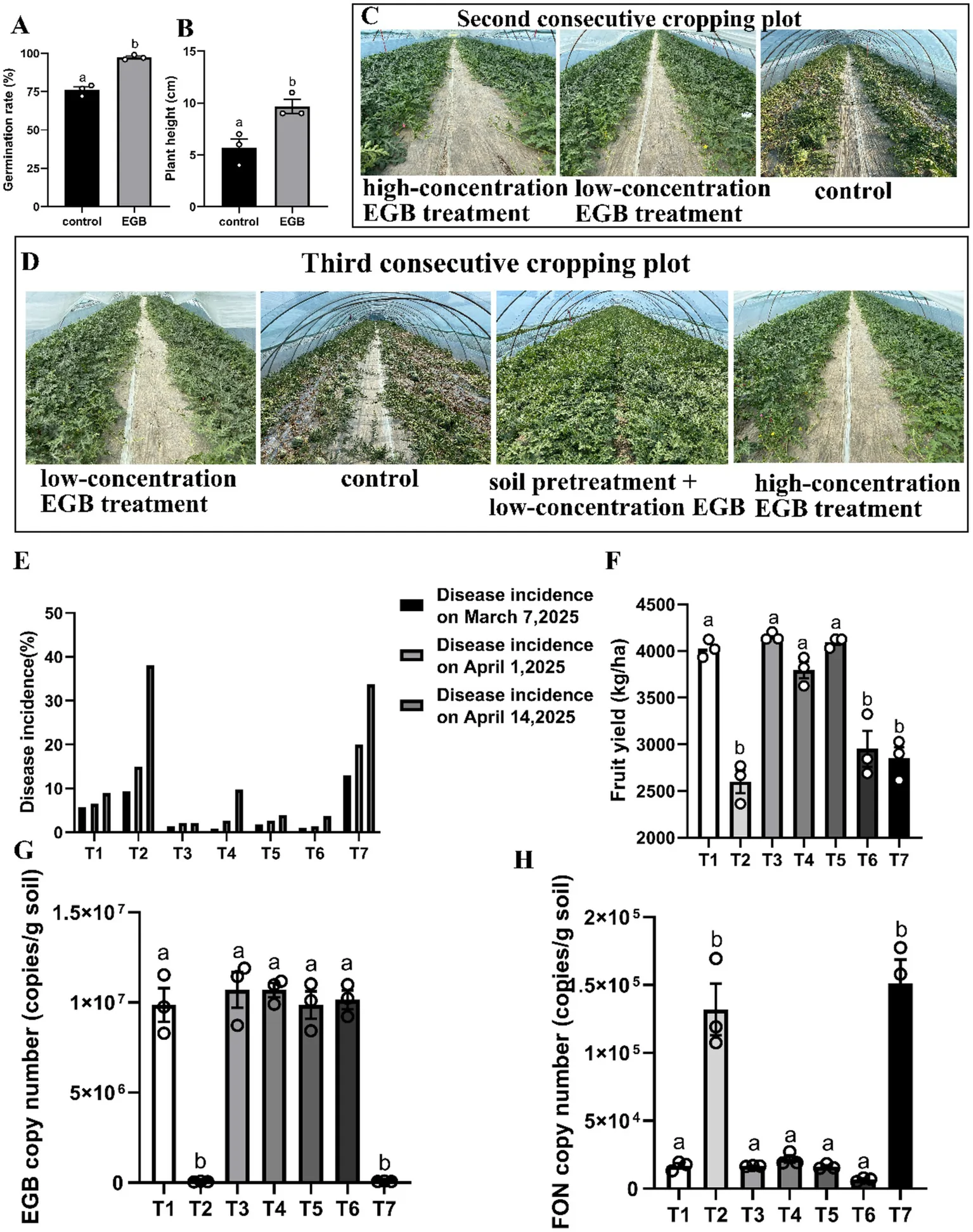
Field biocontrol of watermelon Fusarium wilt by EGB. **(A)** Germination rate of watermelon seeds 15 days after sowing. **(B)** Plant height of watermelon seedlings on the day of transplantation. **(C)** Field growth of watermelon under different treatments in the second consecutive cropping plot. **(D)** Field growth of watermelon under different treatments in the third consecutive cropping plot. **(E)** Temporal changes in disease incidence of watermelon Fusarium wilt under different EGB treatments. Disease incidence was recorded on March 7, April 1, and April 14, 2025, in different field plots (T1–T7). The bars represent disease incidence (%) at each investigation date. **(F)** Fruit yield of watermelon in different plots. **(G)** EGB target gene copy numbers in root-zone soil from different plots. **(H)** FON target gene copy numbers in root-zone soil from different plots. Data are shown as mean ± standard error. Germination rate, main vine length, fruit yield, and EGB copy numbers were analyzed using the Kruskal–Wallis test, followed by Dunn’s multiple comparison test. Different lowercase letters indicate significant differences among treatments (*p* < 0.05).

qPCR analysis showed low copy numbers of myxobacteria in groups T2 and T7, attributing to the low level of non-specific amplification, whereas EGB target gene copy numbers in the EGB-treated plots T1, T3, T4, T5, and T6 were approximately 10^7^ copies g^−1^ root-zone soil, indicating that EGB sustained a high population in field soil (Figure 4G). qPCR analysis further showed that FON target gene copy numbers were relatively high in the control plots T2 and T7, reaching 1.3 × 10^5^ and 1.5 × 10^5^ copies g^−1^ root-zone soil, respectively, whereas FON in EGB-treated plots had lower copy numbers of approximately 10^4^ copies g^−1^ root-zone soil (Figure 4H), which was accompanied by a lower incidence of Fusarium wilt (Supplementary Table S11). These findings indicate that EGB treatments show decrease FON target gene copy numbers and Fusarium wilt incidence.

### 3.5 Effects of EGB treatments on soil bacterial community structure under continuous cropping conditions

Once introduced into soil, strain EGB can prey on soil bacteria and fungi, thereby reshaping the soil bacterial community. To test this hypothesis, we analyzed the diversity and composition of soil bacterial communities in the field experiment using high-throughput sequencing (Figure 5). The α-diversity indices Chao1, ACE, and Shannon were significantly lower in the EGB-treated groups than in the control groups (*p* < 0.05) (Figures 5A–D). Principal component analysis (PCA) showed that PC1 and PC2 accounted for 33.75 and 8.66% of the total variance, respectively (Figure 5E). Along the PC1 axis, PCA revealed a clear separation in bacterial community structure between the control groups (T2 and T7) and the EGB-treated groups (T1, T3, T4, T5, and T6), indicating that EGB application was strongly associated with shifts in bacterial community assembly in continuous-cropping watermelon soil. Consistent with these findings, EGB treatment modified both the ecological strategy composition and the phylum-level structure of the soil bacterial community. Niche breadth analysis indicated that the community was dominated by generalist taxa. Generalists, specialists, and neutral taxa accounted for 67.33, 22.41, and 10.26% of the bacterial taxa, respectively. These results indicate that most bacterial taxa occupy broad ecological niches and may be able to adapt to a wide range of environmental conditions (Figure 5F). Community ecological type analysis showed that persistent taxa contributed a relatively large share of total relative abundance in all treatment groups, whereas intermittent taxa made up a larger share of taxon richness. This pattern indicates the coexistence of stable core microbial groups and numerous low-abundance taxa that fluctuate strongly (Figure 5G). Phylum-level analyses revealed pronounced differences in bacterial community structure among treatments. The dominant phyla were *Pseudomonadota*, *Chloroflexota*, *Actinomycetota*, *Acidobacteriota*, *Gemmatimonadota*, *Planctomycetota*, *Bacillota*, and *Myxococcota*. Relative to the control plots, the EGB-treated plots showed substantial shifts in phylum-level composition, indicating that EGB reshaped the bacterial community structure in watermelon root zone soils under continuous cropping conditions. These compositional shifts may affect soil microbial ecological functions by changing the relative abundance of persistent versus transient taxa (Figure 5H). To further evaluate whether these bacterial community patterns were influenced by the sequence-processing strategy, the sequencing data were additionally analyzed using an ASV-based workflow. The ASV-based analysis showed generally similar treatment-associated patterns to those obtained using the 97% OTU-based approach. In particular, Shannon and Simpson diversity displayed highly comparable trends among treatments, while PCA and phylum-level community composition also showed broadly similar patterns. Although the absolute values of the Chao1 richness estimator differed between the OTU- and ASV-based datasets, the major relative patterns among treatments were generally maintained (Supplementary Figure S2).

**Figure 5:**
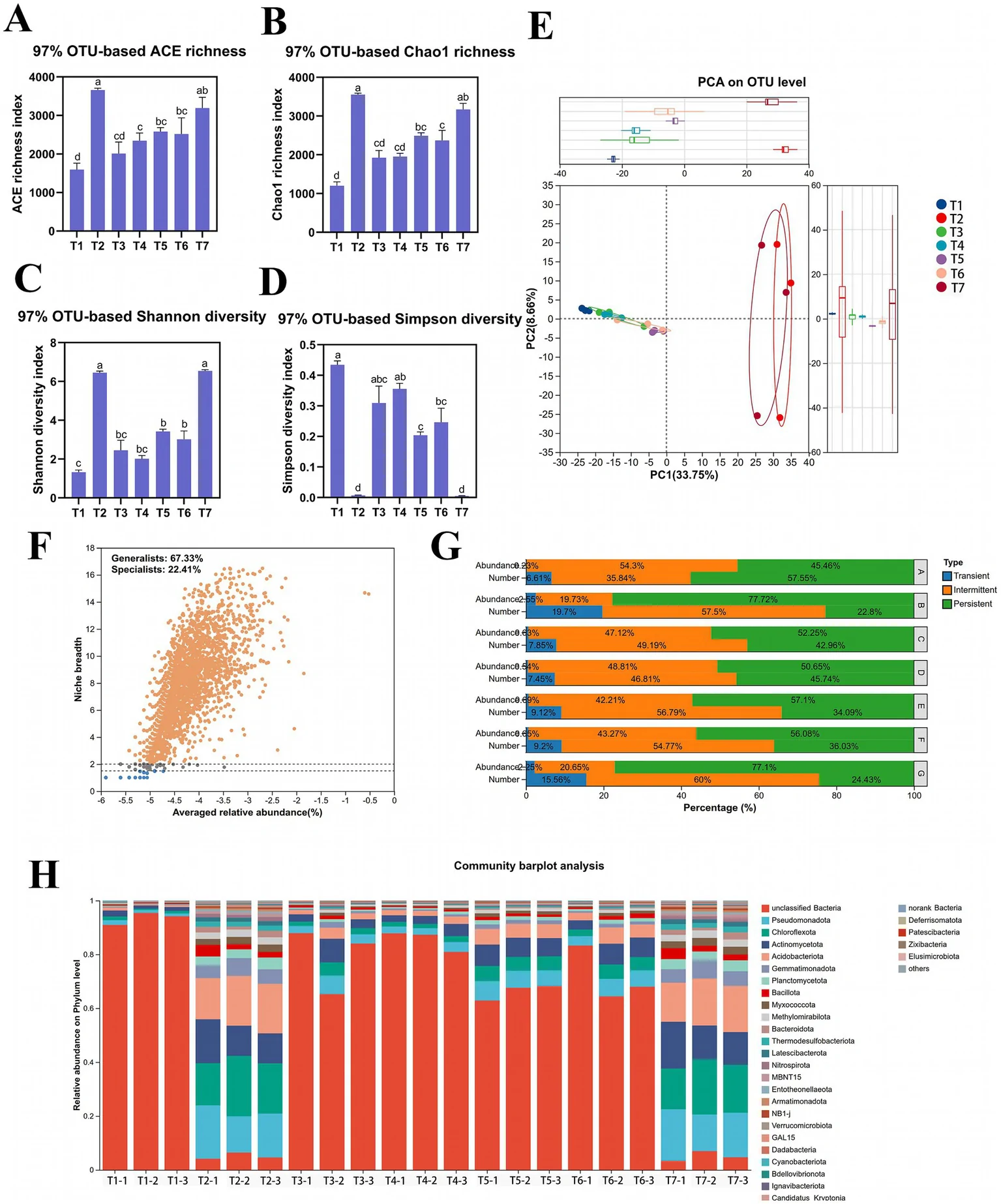
Effects of EGB treatments on bacterial community diversity, community structure, and ecological type composition in watermelon field soil. α-diversity indices of soil bacterial communities in different treatment plots, including the ACE index **(A)**, Chao1 index **(B)**, Shannon index **(C)**, and Simpson index **(D)**. **(E)** Principal component analysis (PCA) based on OTU-level abundance. **(F)** Niche breadth distribution of soil bacterial OTUs. **(G)** Proportions of transient, intermittent, and persistent OTUs in different treatment plots. **(H)** Soil bacterial community composition at the phylum level in different treatment plots. For bar plots, bars represent the mean ± standard error. Different lowercase letters indicate significant differences among treatments (*p* < 0.05).

## 4 Discussion

Watermelon Fusarium wilt remains a persistent threat to watermelon production, particularly in continuously cropped fields where the disease becomes increasingly difficult to control (Rahman et al., 2021; Lv et al., 2025). Once established in soil populations, it can cause extensive plant wilting, severe yield losses, and complete crop failure in extreme cases (Hudson et al., 2021a; Rahman et al., 2021). The long-term persistence of FON largely stems from its capacity to produce chlamydospores, which enable prolonged survival in soil and promote the accumulation of pathogen inoculum over successive cropping cycles (Hudson et al., 2021a; Rahman et al., 2021; Lv et al., 2025). Effective management is further complicated by the physiological race diversity of FON. Among the known races, race 3 is particularly problematic because of its high virulence and its ability to infect cultivars that are resistant to other races, thereby limiting the practical deployment of resistant cultivars in the field (Zhou et al., 2010; Hudson et al., 2021b). In addition, repeated fungicide applications do not fully eliminate soil-borne inoculum and may increase the risk of fungicide resistance and disruption of soil microbial communities (Hudson et al., 2021c; Rahman et al., 2021). Identification of the causal pathogen and its physiological race is essential for evaluating regional disease risk, selecting suitable resistant cultivars, and developing sustainable management strategies. In this study, we characterized a pathogenic *Fusarium oxysporum* f. sp. *niveum* DT0916 isolated from a watermelon Fusarium wilt field in Dongtai, Jiangsu Province, China, which may belong to a putative race 3 isolate based on pathogenicity characteristics and molecular marker analysis. The differential-host reaction pattern did not clearly separate race 3 from race 2, whereas the molecular marker profile was aligned with race 3. This discrepancy may be due to the absence of PI 296341-FR from the host panel and the sensitivity of host responses to experimental conditions. Because race-associated markers may also vary among genetic backgrounds, we treated the two assays as complementary lines of evidence and provisionally designated FON DT0916 as a putative race 3 isolate. However, further validation using PI 296341-FR and authenticated reference isolates is required. Based on these findings, we propose a sustainable disease management strategy that uses the predatory bacterium *Corallococcus* sp. EGB as a biocontrol agent to manage watermelon Fusarium wilt through direct predation and regulation of the soil bacterial community in continuous cropping systems under natural conditions. Our identification of the pathogens responsible for watermelon Fusarium wilt, together with this control strategy, provides a broader understanding of pathogen physiological race differentiation and supports ecological protection in the sequential cultivation of cash crops.

Biological control is widely considered a sustainable strategy for managing soil-borne diseases, but achieving reliable efficacy under field conditions remains a major challenge for many microbial agents (Niu et al., 2020; Ptaszek et al., 2023). *Bacillus, Pseudomonas,* and *Trichoderma* have been extensively studied for the control of Fusarium wilt and related soil-borne diseases; however, their performance is highly dependent on soil properties, rhizosphere conditions, and interactions with resident microbial communities (Weller, 2007; Woo et al., 2023). *Bacillus* spp. are advantageous because they form stress-resistant spores. Their pathogen-suppressive activity is primarily mediated by lipopeptide antibiotics, competition for nutrients and ecological niches, and the induction of plant resistance (Miljaković et al., 2020). However, in continuously cropped soils, high pathogen pressure, intense rhizosphere competition, and altered soil physicochemical properties can reduce their colonization and long-term antagonistic activity (Niu et al., 2020). *Pseudomonas* spp. suppress pathogens by producing siderophores, antibiotics, and volatile compounds, but their persistence and root colonization are often limited by fluctuations in soil moisture, pH, temperature, and competition with indigenous microbes (Weller, 2007). As a result, the strong disease suppression observed in controlled pot or greenhouse experiments does not always translate into reliable field performance (Ptaszek et al., 2023). These limitations highlight the need for biocontrol agents with stronger environmental adaptability, stable colonization, and multiple complementary modes of action in continuous cropping systems. Compared with conventional biocontrol agents, myxobacteria have more complex ecological traits that may be advantageous in continuously cropped soils, where pathogen accumulation is often accompanied by disruption of the resident microbial community (Bull et al., 2002; Zhang et al., 2024). Through gliding motility and coordinated group predation, myxobacteria actively migrate toward nutrient sources or microbial prey and attack target microorganisms. They also secrete extracellular hydrolytic enzymes, antimicrobial metabolites, and outer membrane vesicles that damage fungal hyphae, spores, and cell wall structures (Li et al., 2017; Li et al., 2019; Wang et al., 2025). *Corallococcus* sp. EGB showed biocontrol activity by multifunctional mechanisms, including predation of fungi, rhizosphere migration for colonization, and microbiome regulation for pathogen inhibition. Previous studies have shown that *Corallococcus* sp. EGB suppresses *Fusarium oxysporum*, migrates toward plant roots in soil, reduces pathogen abundance, and reshapes the soil microbial community, thereby enhancing the soil’s disease-suppressive capacity (Li et al., 2017; Ye et al., 2020; Zhao et al., 2021). Such combined of direct and microbiome-mediated effects make EGB particularly promising for managing Fusarium wilt in continuous cropping systems.

Previous studies have shown the biocontrol potential of *Corallococcus* sp. EGB against *Fusarium oxysporum* in plate, pot, greenhouse, and cucumber field systems, but direct field evidence for EGB-mediated control of Fusarium wilt remains limited (Ye et al., 2020; Zhao et al., 2021; Ye et al., 2023). Under field conditions, soil properties are heterogeneous, environmental factors fluctuate, pathogen inoculum is unevenly distributed, and rhizosphere microbial communities change dynamically (Parris et al., 2023). These factors often determine whether a biocontrol agent can successfully colonize the rhizosphere and maintain disease suppression. In this study, EGB was evaluated in a continuously cropped watermelon field. Relatively high EGB target-gene copy numbers were detected under field conditions and were associated with lower FON target-gene copy numbers and reduced Fusarium wilt incidence, indicating the association of EGB with disease suppression in continuous-cropping field systems. Our study extended the biocontrol evaluation of greenhouse experiments to the field, providing direct evidence supporting the potential use of myxobacteria EGB for watermelon Fusarium wilt management.

Stable microbial communities contribute to disease suppression in agricultural systems, while predation impacts prey population dynamics and the broader ecosystem (Hol et al., 2016). Predatory protists have been shown to play a regulatory role in shaping plant growth-promoting rhizobacterial consortia, and predatory myxobacteria also play important roles in shaping microbial communities and recycling of soils nutrients (Petters et al., 2021; Guo et al., 2024). Beyond reducing FON abundance, EGB treatment altered overall community composition, dominant bacterial taxa, and alpha-diversity patterns. These findings indicate that the suppressive effect of EGB is not solely due to direct antagonism or predation against FON, but also associated microbiome-mediated regulation. Although microbial diversity is often considered an important indicator of ecosystem complexity, higher α-diversity does not necessarily correspond to stronger disease resistance (Wagg et al., 2019). EGB application decreased bacterial α-diversity while concurrently reducing FON gene copy numbers and Fusarium wilt incidence, revealing that disease suppression may be associated more with selective changes in bacterial community composition and microbial interactions than with overall microbial richness.

As an introduced biocontrol bacterium, EGB application may alter indigenous microbial assembly through competitive exclusion, resource competition, and niche modification. Notably, the supplementary ASV-based analysis showed treatment-associated patterns broadly consistent with those obtained using the 97% OTU-based approach, particularly for Shannon and Simpson diversity, PCA, and phylum-level community composition. Although absolute richness estimates differed between the two approaches, the major ecological patterns among treatments were generally maintained. Such differences are not unexpected because ASV inference and 97% OTU clustering differ in feature resolution and sequence denoising, which can particularly affect richness estimators that are sensitive to rare features. Such ecological filtering may reduce some less competitive or functionally redundant taxa while favoring bacterial groups potentially associated with pathogen suppression. Thus, the observed decrease in α-diversity may reflect a shift toward a more selectively structured bacterial community rather than a simple loss of ecological function (Louca et al., 2018; Mallon et al., 2018; Ye et al., 2020). Together with the field colonization of EGB, the decrease in FON copy number, and the reduction in disease incidence, these observations support a multi-component mode of action in which EGB suppresses watermelon Fusarium wilt through direct pathogen inhibition, root zone establishment, bacterial community restructuring, and reinforcement of soil suppressiveness. Therefore, EGB may act not only as a pathogen-targeting biocontrol agent, but also as a microbiome-modulating agent that enhances the ecological resilience of continuously cropped soils. Future studies integrating bacterial and fungal community profiling with functional analyses may provide a more comprehensive understanding of the microbial interactions underlying EGB-mediated suppression of watermelon Fusarium wilt.

## 5 Conclusion

This study characterized FON DT0916 from a continuously cropped watermelon field as *Fusarium oxysporum* f. sp. *niveum* and provisionally assigned it as a possible race 3 isolate. We further demonstrated that EGB application was associated with lower FON target gene copy numbers and reduced disease incidence under field conditions. High-throughput sequencing indicated that EGB-mediated disease suppression likely arises from both direct inhibition of the pathogen and reshaping of the bacterial community in continuously cropped soil. These findings provide additional insights into the occurrence and characterization of a putative FON race 3 isolate in Dongtai, Jiangsu Province, and provide field-based evidence supporting the potential application of biocontrol myxobacteria for watermelon Fusarium wilt management. The observed changes in soil bacterial community structure following EGB application suggest that microbial community shifts may contribute to disease suppression under field conditions. However, FON DT0916 was provisionally assigned to race 3 based on the available molecular evidence. Further testing using PI 296341-FR or another validated differential host is required to confirm its physiological race. Multi-season and multi-location trials are still needed to evaluate the long-term stability of EGB and its applicability across different watermelon-producing regions and soil types.

## Data Availability

The original contributions presented in the study are included in the article/Supplementary material, further inquiries can be directed to the corresponding authors.
