# Supplementary material for: Biocontrol of watermelon Fusarium wilt caused by Fusarium oxysporum f. sp. niveum with Corallococcus sp. EGB
Source: Front Microbiol. 2026 Sep 17;17:1950692. doi: 10.3389/fmicb.2026.1950692 (PMC13629182; doi:10.3389/fmicb.2026.1950692)
Supplement: Supplementary file 1 [file Data_Sheet_1.PDF]

Supplementary Table 1. Primer sequences used for molecular identification and physiological race detection of FON DT0916

primer	sequence
TEF-1α	5’-CATCGAGAAGTTCGAGAAGG-3’
	5’-TACTTGAAGGAACCCTTACC-3’
β-tubulin	5’-GGTAACCAAATCGGTGCTGCTTTC-3’
	5’-ACCCTCAGTGTAGTGACCCTTGGC-3’
ITS1	5’-TCCGTAGGTGAACCTGCGG-3’
ITS4	5’-TCCTCCGCTTATTGATATGC-3’
W106	5’-GCAGTCGTACGTCATCGACC-3’
	5’-CCA TGGCAGATGGCGAGTCA-3’
FOW-1	5’-TCAGTCCTGGATAGTTTGGTGT-3’
	5’-GCGTTGTTCATAGATGCGTTAGT-3’
SIX6	5’-ATGAAGCTCGCTCTTATCGCATC-3’
	5’-AGAACTGGAAAAGGTCGCTGC-3’
FNR3	5’-CGGCTTTCCTCTGTCAGATAGT-3’
	5’-TAGTGAGGTCCATGCCACGAA-3’

Supplementary Table 2. Cropping history, disease background, and baseline soil physicochemical properties of the experimental field

Category	Parameter	Value/description
Field background	Experimental-field consistency	All treatments were established within the same continuous watermelon-cropping field
Cropping history	Continuous watermelon cultivation	2 years
Disease background	Historical incidence of Fusarium wilt	>30% in each year
Disease background	Field classification	Field with a history of severe watermelon Fusarium wilt
Soil property	Organic matter	21.4 g kg^{-1}
Soil property	Available nitrogen	85.6 mg kg^{-1}
Soil property	Available phosphorus	34.2 mg kg^{-1}
Soil property	Available potassium	120.3 mg kg^{-1}
Soil property	pH	6.8

Supplementary Table 3. Cropping history and dimensions of the experimental field plots.

Plot	Cropping history	Plot dimensions (m)	Plot area (m^2)
T1	Third consecutive cropping	47.9 × 6.0	287.4
T2	Third consecutive cropping	47.9 × 6.0	287.4
T3	Third consecutive cropping	47.9 × 6.0	287.4
T4	Third consecutive cropping	47.9 × 6.0	287.4
T5	Second consecutive cropping	47.9 × 6.0	287.4
T6	Second consecutive cropping	47.9 × 6.0	287.4
T7	Second consecutive cropping	18.2 × 12.0	218.4

Supplementary Table 4. Field application schedule of EGB

Application	Growth stage	Date	Application description
First	Transplanting	February 13, 2025	EGB was applied once with the transplanting water on the day of field transplantation.
Second	Vine development stage	March 8, 2025	EGB was applied during the vine development stage.
Third	Pre-pollination stage	March 26, 2025	EGB was applied once before pollination.
Fourth	Early fruit-setting stage	April 7, 2025	EGB was applied during the early fruit-setting stage.
Fifth	Late fruit-setting stage	April 14, 2025	EGB was applied during the late fruit-setting stage.

Supplementary Table 5. Timeline of the field experiment

Event	Date
Incorporation of EGB into the seedling substrate and seed sowing	January 9, 2025
Field transplantation and first EGB application	February 13, 2025
First disease incidence assessment	March 7, 2025
Second EGB application	March 8, 2025
Third EGB application	March 26, 2025
Second disease incidence assessment	April 1, 2025
Fourth EGB application	April 7, 2025
Third disease incidence assessment	April 14, 2025
Fifth EGB application	April 14, 2025
Harvest and soil sampling for EGB and FON detection and high-throughput sequencing of the soil microbial community	May 12, 2025

Supplementary Table 6. Primer information used for qPCR quantification

Target	Primer Sequence (5′–3′)	Target gene	Notes
EGB	EGB-F TCATCATCGGCACTGTCATC	TonB-dependent receptor gene	Single-copy gene; primers designed based on comparative genomic analysis of *Corallococcus* sp. EGB and other myxobacteria
EGB	EGB-R GGATGGTGCGGTTGAGGAGC	TonB-dependent receptor gene	Single-copy gene
FON	FON-F GTTGCTTACGGTTCTAACTGTGC	Mitochondrial cytochrome c oxidase subunit II (*cox2*) gene	Specific for *F. oxysporum* f. sp. *niveum* strain Fon005
FON	FON-R GGTACTTGGAAGGAATTGTGGG	Mitochondrial cytochrome c oxidase subunit II (*cox2*) gene	Specific for *F. oxysporum* f. sp. *niveum* strain Fon005

Supplementary Table 7. qPCR reaction system and thermal cycling conditions

Parameter	Condition
qPCR reagent	Taq Pro Universal SYBR qPCR Master Mix (Vazyme)
Instrument	QuantStudio™ 6 Flex Real-Time PCR System
Total reaction volume	20 μL
qPCR Master Mix	10 μL
Forward primer	0.4 μL
Reverse primer	0.4 μL
Template DNA	2 μL
Nuclease-free water	7.2 μL
Initial incubation	50 °C for 2 min
Initial denaturation	95 °C for 10 min
Amplification	40 cycles
Denaturation	95 °C for 15 s
Annealing/extension	60 °C for 34 s
Quantification output	Target gene copies per gram of soil

Supplementary Table 8. Bioinformatics pipeline for 16S rRNA gene sequencing data

Step	Software / Database	Version	Parameters / Description
Quality filtering	fastp	0.23.4	Raw paired-end reads were quality filtered
Read merging	FLASH	1.2.11	Paired-end reads were merged based on overlapping regions
Dereplication	UPARSE workflow	—	Optimized sequences were dereplicated and singleton sequences were removed
OTU clustering	UPARSE	7.0.1090	OTUs were clustered at 97% sequence similarity
Chimera removal	UPARSE	7.0.1090	Chimeric sequences were removed during OTU clustering
OTU table generation	UPARSE	7.0.1090	Quality-filtered sequences were mapped to representative OTU sequences at ≥97% similarity
Taxonomic classification	RDP Classifier	2.11	Representative OTU sequences were classified with a confidence threshold of 0.7
Reference database	SILVA	Release 138	Used for taxonomic assignment
Alpha-diversity analysis	Mothur	1.48.3	Used to calculate alpha-diversity indices
Taxonomic abundance summaries	QIIME	1.9.1	Used for taxonomic abundance summaries
Beta-diversity analysis	QIIME	1.9.1	Used for beta-diversity analyses

Supplementary Table 9. Ecological classification and statistical analysis of bacterial OTUs

Analysis	Method / Software	Definition / Threshold	Statistical test
Niche breadth	spaa package in R	$B_i = 1/\sum_{j=1}^{n} p_{ij}^2$	Two-sided Wilcoxon rank-sum test
Software environment	R 3.3.1; spaa 0.2.2	OTU-level calculation	-
Prevalence filtering before niche-breadth analysis	None	Prevalence threshold = 0	-
OTU prevalence	Proportion of samples in which an OTU was detected	-	-
Transient taxa	Prevalence-based classification	Prevalence < 0.2	-
Intermittent taxa	Prevalence-based classification	0.2 ≤ prevalence ≤ 0.8	-
Persistent taxa	Prevalence-based classification	Prevalence > 0.8	-

Supplementary Table 10. Replicate-level disease index data from the pathogenicity assay

Treatment	Pot 1	Pot 2	Pot 3	Pot 4	Pot 5	Pot 6	Pot 7	Mean ± SEM (%)
CK	0	0	0	0	0	0	0	0.0 ± 0.0
FON DT0916	50.0	25.0	83.3	91.7	58.3	50.0	91.7	**64.3 ± 9.6**

Supplementary Table 11. Disease incidence of watermelon Fusarium wilt in different field plots at different investigation dates.

Plot code	Total number of plants	Disease incidence on March 7, 2025	Disease incidence on April 1, 2025	Disease incidence on April 14, 2025
T1	383	5.74%	6.53%	8.88%
T2	383	9.40%	14.88%	38.02%
T3	383	1.31%	2.09%	2.09%
T4	383	0.78%	2.61%	9.75%
T5	383	1.83%	2.61%	3.92%
T6	383	1.04%	1.31%	3.66%
T7	291	13.40%	19.93%	33.68%

Supplementary Figure 1. Effects of EGB application on main vine length of watermelon under continuous-cropping conditions.

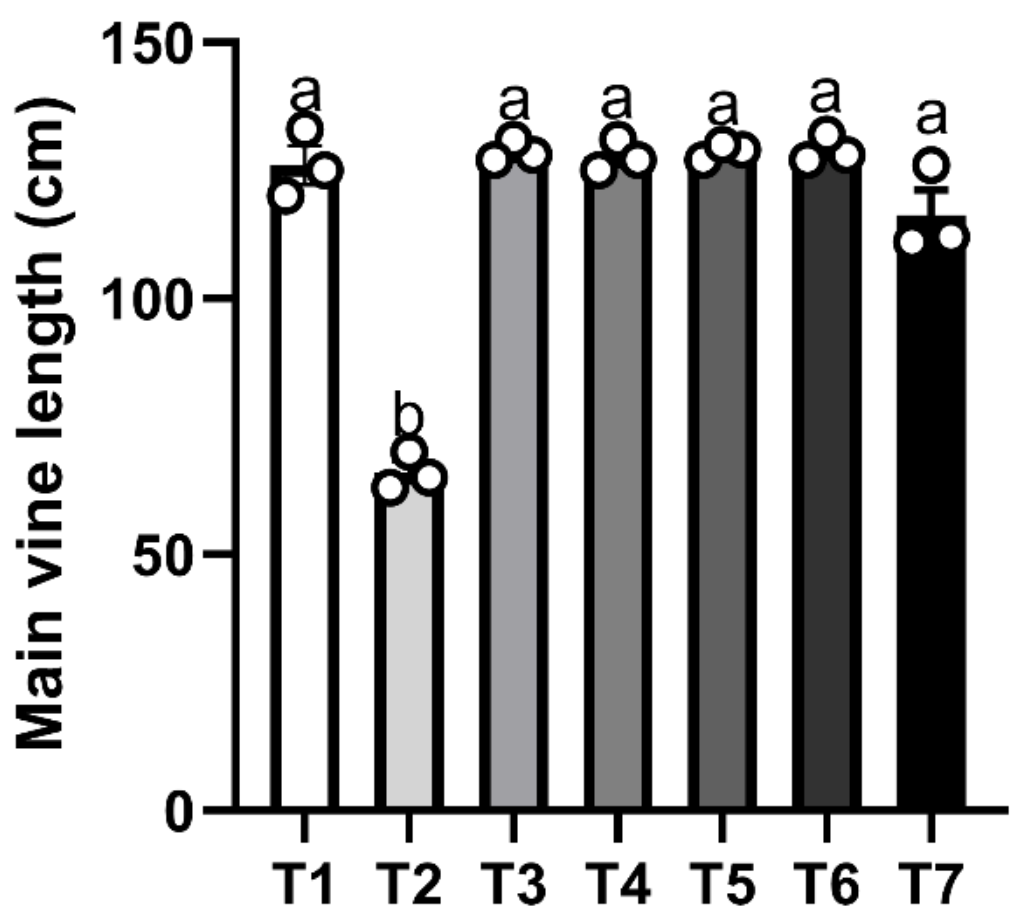

Supplementary Figure 1. Effects of EGB application on the main vine length of watermelon under continuous-cropping conditions. Main vine length was measured in plants subjected to treatments T1–T7. Data are presented as mean ± SEM. Different lowercase letters indicate significant differences among treatments according to one-way ANOVA followed by Tukey's multiple-comparison test ($p < 0.05$).

Supplementary Figure 2. Comparison of bacterial community analyses based on 97% OTUs and ASVs.

97% OTU-based analysis

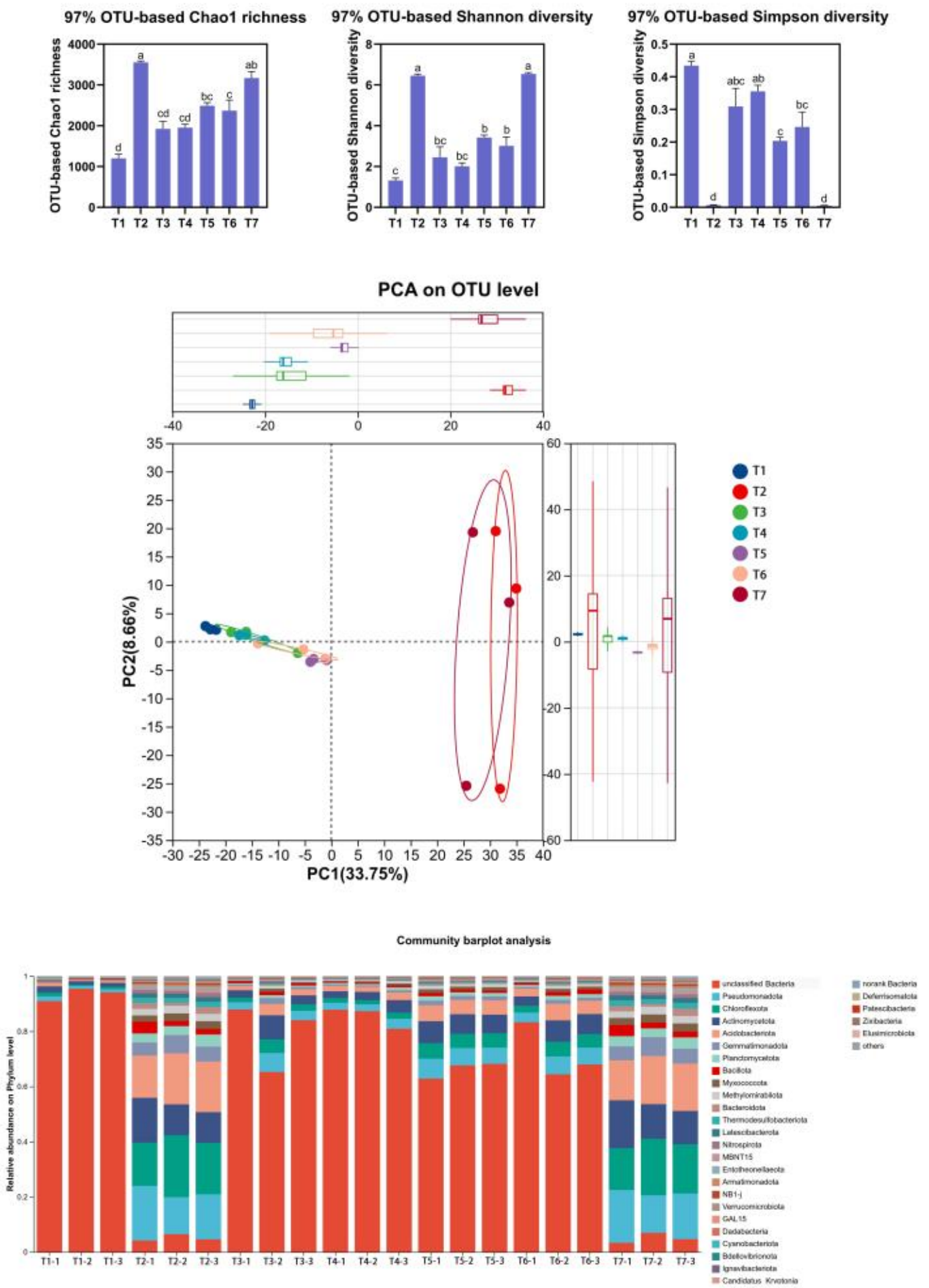

ASV-based analysis

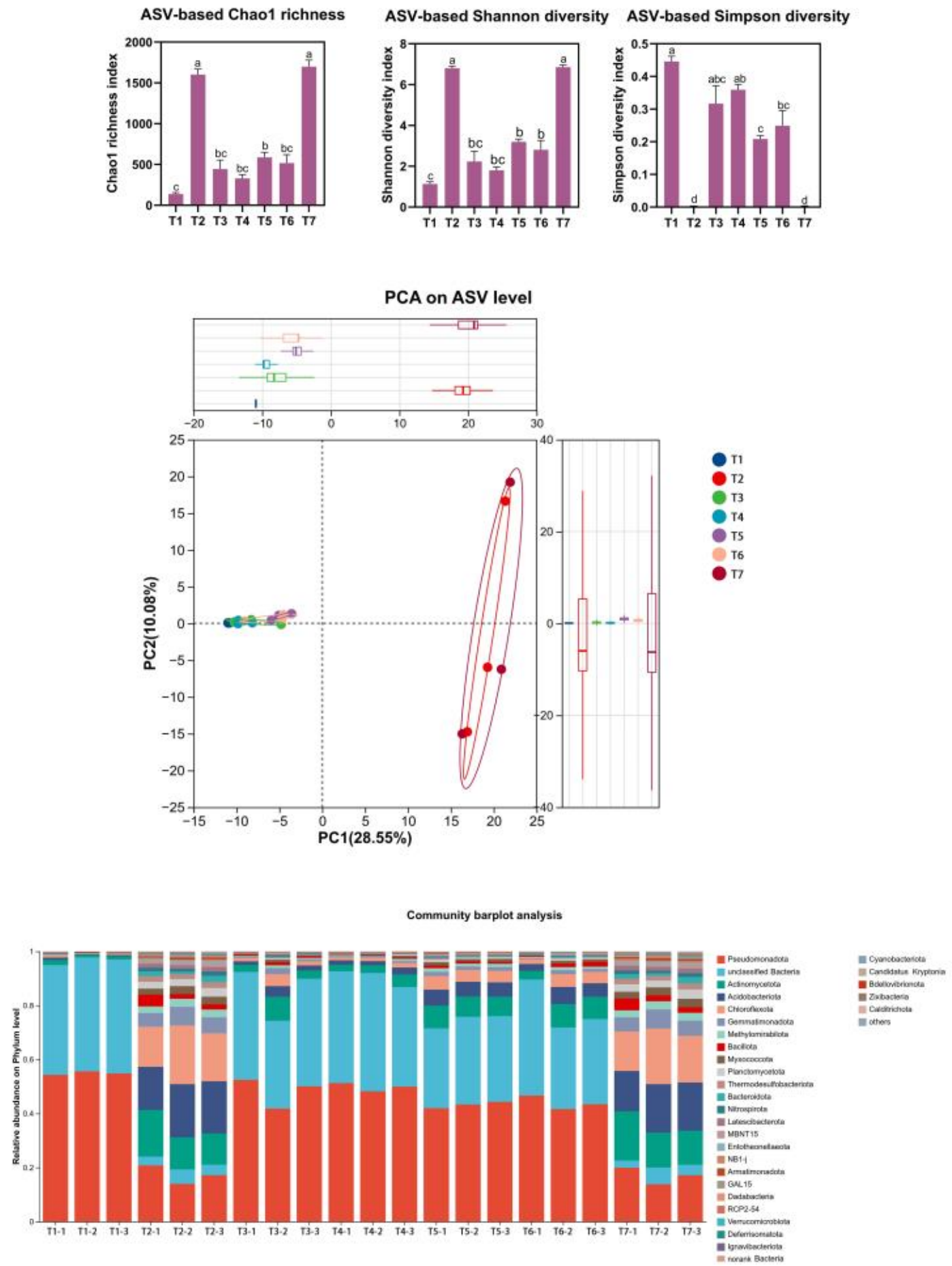

Supplementary Figure 2. Comparison of bacterial community patterns obtained using 97% OTU-based and ASV-based analyses. Alpha diversity was evaluated using the Chao1, Shannon, and Simpson indices. Community variation was visualized by principal component analysis (PCA), and bacterial community composition was compared at the phylum level. Overall, the ASV-based analysis showed patterns broadly consistent with those obtained using the 97% OTU-based approach.
